# State-of-the-Art Review on Immersive Virtual Reality Interventions for Colonoscopy-Induced Anxiety and Pain

**DOI:** 10.3390/jcm11061670

**Published:** 2022-03-17

**Authors:** Marcel-Alexandru Găină, Andreea Silvana Szalontay, Gabriela Ștefănescu, Gheorghe Gh Bălan, Cristina Mihaela Ghiciuc, Alexandra Boloș, Alexandra-Maria Găină, Cristinel Ștefănescu

**Affiliations:** 1 Psychiatry, Department of Medicine III, Faculty of Medicine, Grigore T. Popa University of Medicine and Pharmacy of Iasi, 16 Universitatii Street, 700115 Iasi, Romania; andreea.szalontay@umfiasi.ro (A.S.S.); alexandra.bolos@umfiasi.ro (A.B.); cri.stefanescu@umfiasi.ro (C.Ș.); 2 Institute of Psychiatry “Socola”, 36 Bucium Street, 700282 Iasi, Romania; 3 Medical Semiology and Gastroenterology, Department of Medicine I, Faculty of Medicine, Grigore T. Popa University of Medicine and Pharmacy of Iasi, 16 Universitatii Street, 700115 Iasi, Romania; gabriela.stefanescu@umfiasi.ro (G.Ș.); gheorghe-g-balan@umfiasi.ro (G.G.B.); 4 2nd Gastroenterology Ward, Saint “Spiridon” County Hospital, Independence Bvd. No 1, 700111 Iasi, Romania; 5 Pharmacology, Clinical Pharmacology and Algeziology, Department of Morpho-Functional Sciences II, Faculty of Medicine, Grigore T. Popa University of Medicine and Pharmacy of Iasi, 16 Universitatii Street, 700115 Iasi, Romania; cristina.ghiciuc@umfiasi.ro; 6 1st Neurology Ward, Hospital of Neurosurgery “Prof. Dr. Nicolae Oblu” Iasi, 2 Ateneului Street, 700309 Iasi, Romania; alexandra-maria.popescu@d.umfiasi.ro

**Keywords:** virtual reality, mental health, anxiety, pain management, colonoscopy screening, VR training, VR education, consumer VR

## Abstract

Background: Colonoscopy related fear impairs the current gold standard screening of colorectal cancer. Compared to other minimally invasive procedures for cancer screening, colonoscopy-induced anxiety exceeds the procedure through bowel preparation. Immersive virtual reality’s (iVR) role in alleviating the complex stress–pain relationship encountered during medical procedures is directly proportional to the rising affordability of state-of-the-art Head-Mounted-Displays (HMDs). Objective: to assess the effect of iVR on patients’ colonoscopy-induced anxiety and pain. Materials and methods: A systematic search was conducted in PubMed, Cochrane Central Register of Controlled Trials, Web of Science, Embase and Scopus databases up to January 2022. Clinical trials evaluating anxiety as an outcome were included without language restriction. Results: Four clinical trials were included: three on the patients’ intraprocedural anxiety and one on patient education. Intraprocedural iVR interventions for colonoscopy-induced anxiety and pain revealed a similar effect as conventional sedation, while a statistically significant reduction was reported for non-sedated patients. iVR patient education improved the quality of bowel preparation and reduced patient anxiety before colonoscopy. Conclusions: The current research highlights the need to use high-end HMDs and appropriate interactive iVR software content for colonoscopy-induced anxiety. Methodological frameworks regarding the eligibility of participants, double-blinding and randomization of iVR studies can facilitate the development of iVR implementation for anxiety and pain management.

## 1. Introduction

Colonoscopy still stands as the gold standard for colorectal cancer (CRC) screening and premalignant lesions worldwide in terms of sensitivity and specificity, although it is anticipated with fear. Regarding factors that influence patients’ failure to attend a scheduled colonoscopy, the most significant barrier corresponds to the emotional and cognitive manifestations of the fear of cancer, which is common in up to 45.2% of CRC screening candidates [1]. Risk populations, such as inflammatory disease patients, consider colonoscopy invasive, uncomfortable, potentially painful, and embarrassing [2]. Shafer et al., 2018, reported high anxiety scores in patients undergoing bowel preparation and colonoscopy: related to the procedure (29%), related to the bowel preparation (18%), and about the results (28%) [3]. A comparative sedated versus non-sedated colonoscopy clinical trial showed higher anxiety levels correlated with a greater need for sedation and lesser willingness to undergo future recommended colonoscopies without conventional sedation [4].

Continuous research efforts are targeting a wider uptake of the CRC screening [5] through finding alternative, currently accepted, non-invasive screening methods [6] because CRC is still accountable for half of the cancer-related deaths worldwide [7]. Colonoscopy is both an exploratory and interventional procedure. It, therefore, may remain a primary screening procedure for CRC. However, other research directions pursue raising sensitivity and specificity of currently available alternative screening procedures, such as the imagistic analysis [8], epigenetic faecal biomarkers [9], urine, and exhaled breath or blood-based biomarkers [10]. In Europe, Colon Capsule Endoscopy is currently considered acceptable as a screening method regarding specificity and sensitivity. Still, its disadvantage comes from higher costs and lack of biopsy sampling [11].

One of the traits of negative outcome during and after minimally invasive diagnostic procedures is represented by a greater perceived pain intensity that is usually related to the coexisting level of procedure-induced anxiety [12]. The colonoscopy procedure is associated with anxiety and pain in an interdependent manner. Therefore, efforts are made to limit these adverse events [13]. In search for improvement of colonoscopy, transdisciplinary views emerge regarding targeting the psychological aspects of the intervention. Dr. Hassan Cesare edifyingly states: “The simplest solution to this problem is to recognise it for what it is: psychology! The personal beliefs, fears, and expectations of patients affect their experiences before, during, and after the examination. Only if we focus on psychological factors can we improve the patient’s experience. This means extending education about colonoscopy to encompass more than the technical details of the procedure and including all the possible stimuli that may affect patient reactions” [14]. Immersive virtual reality (iVR) can represent a disruptive technology in colonoscopy by creating transdisciplinary bridges throughout different specialities to transcend towards individualised interventions improving the quality of patient-related outcomes.

**Figure 1:**
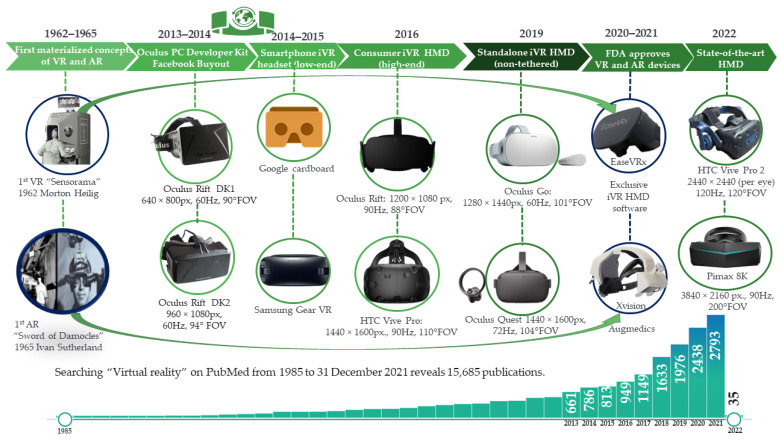
Virtual reality (VR) equipment and yearly publication trends from 1985 to 2022.

The concepts behind virtual reality (VR) and augmented reality (AR) have been intuited since the very dawn of processing units. The technical evolution and growing accessibility of consumer VR equipment are directly proportional to medical research publication trends (Figure 1). Even throughout the SARS-CoV-2 pandemic, technological development continued respecting the doubling number of microprocessors transistors, sensor development, and pixilation density every two years, as stated in the half-century-old Moore’s Law [15], thus facilitating the iVR concept materialisation. The specifications of iVR headsets displays can be simplified as resolution or pixel density of their lenses (px), the field of view (FOV), and refresh rate per second (Hz) [16]. Millstones in the evolution of iVR equipment were made both on AR and VR:

- AR: In 1966, Ivan Sutherland imagined an “ultimate display”, but the lack of graphical processing power determined a resemblance of a primitive form of AR through the first Head Mounted Display (HMD) [17]. Sutherland’s device was backed by a large and heavy computer and mechanism that provided its invention with the name “Sword of Damocles” [18]. Then, 54 years later, on 11 June 2020, Xvision Augmedics AR HMD was approved by the United States Food and Drug Administration (U.S. FDA) for intra-operative use, offering real-time visualisation of individual anatomy of the patient reconstructed from previous scans and therefore facilitating precision in screw insertion [19].
- VR: Through “Sensorama”, Morton Heilig created in 1962 the first interactive, immersive VR (iVR) all-in-one system, meant to capture all senses of the user, from dynamic visuals, auditory, olfactive, and even vibratory sensation [20]. In November 2021, U.S. FDA authorised EaseVRx, a remote cognitive-behavioural approach targeting chronic back pain delivered through an iVR HMD [21].

Although only experimental, emerging deep learning technology can autonomously enhance current screening algorithms improving CRC diagnosis and prognosis [22]. The rising applicability of iVR in medicine is consecutive to exponential technological growth and availability, and it is predicted to become as ubiquitous as smartphones are today [23]. Immersive virtual reality (iVR) interventions are reported to lower patients’ fear concerning a variety of medical procedures such as dental, magnetic resonance claustrophobia, or needle puncture [24], as well as decreasing acute procedural pain [25] and anxiety of both children and adults [26,27,28]. Nowadays, iVR equipment is a more affordable technological breakthrough, reflecting current consumer HMDs higher specifications and lower prices. Beyond pain distraction, recent interventional iVR colonoscopy studies aim to offer an alternative to conventional sedation by targeting common psychological symptoms such as anxiety.

This study aims to assess the effect of iVR on the colonoscopy-induced anxiety of patients undergoing bowel preparation and colonoscopy, either by the distraction of the patient during the procedure or by education for bowel preparation. We hypothesise that a holistic approach may offer a better perspective for previsioning over the current feasibility and future direction of iVR in colonoscopy.

## 2. Materials and Methods

A comprehensive search strategy following the Preferred Reporting Items for Systematic Reviews and Meta-Analyses (PRISMA) [29] guidelines for reporting was used to identify all published work.

### 2.1. Electronic Searches

The literature was searched independently by two authors, up to the 1st of January 2022, using the following electronic databases: PubMed, Cochrane Central Register of Controlled Trials, Web of Science, Scopus, and Embase. Due to the limited number of papers available, there were no specific language restrictions, publication dates, or publication status. We also hand-searched relevant conference abstracts or proceedings, all the references of included articles, and the grey literature from Google Scholar.

The following medical subject headings (MeSH) search terms were defined (Table 1):

Two groups of authors (M.-A.G., G.G.B. and A.-M.G.; C.M.G., A.B. and G.Ș.) independently screened studies and were selected for full-text review. Disagreements regarding the included studies were resolved by discussion and consensus. The third group of authors (A.S.S. and C.Ș.) acted as arbitrators when an agreement was not reached.

### 2.2. Study Selection

Clinical trials evaluating iVR for colonoscopy-induced anxiety were included without language restriction. Outcomes were an assessment of the influence of the intervention on the anxiety and/or pain of the participants. Types of participants: adults (>18 years of age) undergoing colonoscopy, with no restriction regarding gender. Types of interventions: any iVR intervention (iVR regarded as stereoscopic emulated software content through an HMD) with therapeutically or educational potential to lower colonoscopy-induced anxiety (intervention group), compared with the control group with conventional procedure (sedated or non-sedated) or traditional means of education for bowel preparation. We excluded studies that did not share the aforementioned characteristics through a full-text evaluation.

## 3. Results

**Figure 2:**
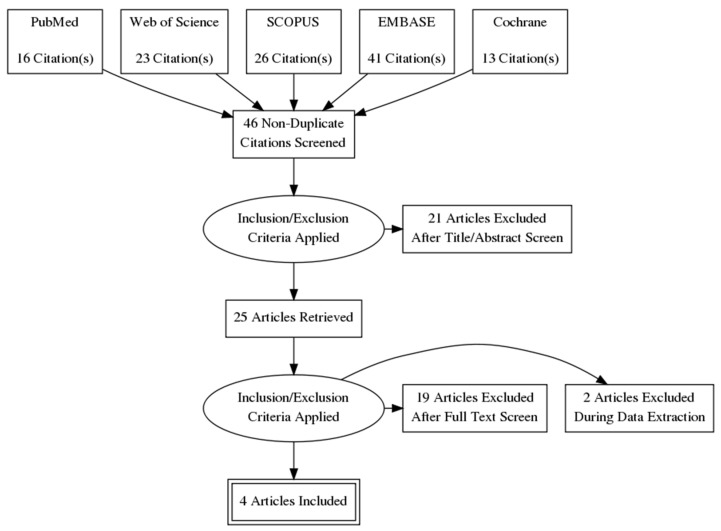
PRISMA flowchart.

Four iVR clinical trials were included (Figure 2): three studies on patients’ intraprocedural anxiety and one on patient education.

### 3.1. Virtual Reality as a Therapy for Reducing the Anxiety of Patients during a Colonoscopy Procedure

Only three interventional studies (Table 2) investigated the effect of iVR HMD distraction on the periprocedural impact on anxiety and pain.

Data extracted in Table 2 reveals that although all authors encountered statistically significant differences in pain perception, the level of significance was not fulfilled regarding lowering anxiety through iVR. None of the studies reported iVR intervention as the potential technical impediment regarding colonoscopy procedure, while patients in VRGs had an overall positive perception regarding the intervention. One of the main limitations of all studies is low-end iVR HMD. All three existing literature studies used more affordable HMD alternatives such as Google Cardboard, and two used Samsung Gear VR.

Veldhuijzen et al., 2020 (Table 2) designed an iVR vs. standard colonoscopy RCT and reported strictly using only a visual iVR experience to distract the patients. During colonoscopy, audio stimulation was eliminated from securing a viable verbal communication channel between the patient and the performing physician. The possibility of managing distraction of participants strictly visually, through a currently discontinued Cardboard headset powered by a 2016 smartphone, was reported to have a low influence on anxiety scores. Participants tested the headsets one day before the study in an initial exposure. Still, there was no precise specification of whether the first initial use of the VR glasses included the sound. Although the prior exposure of the iVR group participants to the immersive virtual experience may represent an excellent manner to reduce the additional anxiety regarding a stressful procedure, a second intraprocedural iVR exposure without sound may mean a less satisfying version of the primary preliminary direction. Nevertheless, preserving the patient’s ability to listen and respond implies focusing the patient’s attention toward the same procedure room. These aspects may have influenced results, explaining the lack of statistical impact of the intervention on the reported median anxiety score, the similarities regarding STAI-T between both groups, and the equally increased anxiety in both Virtual Reality Intervention Group (VGR) and Control Group (CG) The primary aim of iVR acceptability of patients undergoing colonoscopy was achieved. In this study, patients with epilepsy neurosensorial deficits such as auditory, visual, or balance were excluded. Still, among psychiatric disorders, only dementia diagnosed patients were excluded, with no specification regarding the exclusion of patients diagnosed with concurrent anxiety disorders.

Çakır and Evirgen, 2021 (Table 2) reported a significant difference regarding pain perception in the VRG. Still, no significant effect was reported on alleviating anxiety, even if patients diagnosed with psychiatric illness or neurosensorial disorders were excluded. Moreover, the same physician performed all procedures in both groups to reduce possible procedural errors when using iVR. The lack of a high-end iVR HMD was probably compensated by using a dedicated virtual reality relaxation software called “A walk on the beach”, created by Dr. Eric Fassbenders in 2015 [31].

The pilot study design by Friedman et al., 2021 had a different approach to iVR HMD exposure. He compared the same group of participants who underwent at least one colonoscopy under conscious sedation within the same health facility to evaluate the impact of iVR on procedural anxiety and pain. The exclusion criteria only addressed aspects related to the previous procedure, such as having a prior colonoscopy non-sedated or under anaesthesia, with no regard to the participant’s history of psychiatric or neurological disorders. Among the 26 that finished colonoscopy with iVR, six patients (23.1%) asked for medication during the procedure. Still, they resisted temptation as the pain was not perceived as intense enough or after being reassured by the physician that cramping was over. The results showed that the one participant that requested medication had the lowest score of 1 (weakest motivation) to the question regarding the cause for using iVR as an alternative to sedation, compared with the other 26 participants that completed iVR colonoscopy without requesting medication averaging scores in the range of 8.5–9 out of 10. Therefore, they suggest that a strong motivation for choosing iVR might be related to the patient’s determination to avoid sedation medication [32].

### 3.2. iVR Education Reduces Anxiety of Patients before Colonoscopy

Chen et al., 2021, analysed the effects of either conventional education or iVR education for colonoscopy bowel preparation. This 2-year prospective RCT study population included 346 randomly assigned (1:1) to one form of education. The outcomes included compliance, preparation, satisfaction, and detection rates. The authors reported a statistically significant improvement in preprocedural anxiety (Table 3). Anxiety was evaluated indirectly by self-rated sleep quality scores before the procedure (mean, 7.60 vs. 7.08; *p* = 0.04) in the iVR group. Higher satisfaction (mean, 8.68 vs. 8.16; *p* = 0.01), a greater compliance rate (68.8% vs. 50.3%; *p* < 0.001), and an improvement of detection rate for polyps (41.9% vs. 26.7%; *p* = 0.003) and adenomas (32.6% vs. 22.1%; *p* = 0.03) was also reported in the iVR group. The high educational, economic and social status of included patients limit the generalisation of this study results for the general population [33].

## 4. Discussion

The included studies used low-end HMD, which determined questionable results on colonoscopy-induced anxiety. The current study highlights the need to reduce methodological flaws to improve iVR effectiveness in lowering anxiety and pain during colonoscopy.

There is a need to evaluate the iVR impact on anxiety during colonoscopy of high-end dedicated immersive HMD compared with smartphone-powered HMDs, as high-end technology should offer a greater sense of presence and perhaps distractibility during the colonoscopy procedure. For intraprocedural distraction, we emphasise the need for state-of-the-art HMD devices with high-end specifications such as an Oculus Rift or HTC Vive-Pro to offer strong enough variables in the dissociation equation consisting of the potential of immersivity (hardware and software) multiplied by software potential of distractibility. Existing studies demonstrate the superiority of higher immersivity in lowering anxiety and pain compared with non-immersive VR in paediatric populations bone pin and suture removal [34]. As iVR environments become more and more vivid, facilitating a deeper perspective of presence needed for distracting patients undergoing minimally invasive procedures from the “white coats” and somewhat unknown or unfriendly perception of the medical equipment specific to a procedure room. This aspect could be improved by directly bridging with psychotherapists to offer patients dedicated therapy and VR exposure. A guided iVR intraprocedural experience targeting anxiety regarding colonoscopy through a cognitive-behavioural facilitated orientation can influence the third variable of the equation.

The protocol of Çakır and Evirgen, 2021, was submitted to evaluate a Non-Interventional Clinical Research Ethics Committee [31]. Friedman et al., 2021 [32] eluded an Ethical Committee approval, motivated by the exclusion of participants with contraindications to conscious sedation, to ensure the possibility of eventual pharmacological rescue. As an emerging technology, iVR may be perceived as non-interventional. Still, that very perception may have a role in limiting progress through creating an ethical framework preliminary to the standardisation of study designs. The commentary article of Christian Dualé, 2021 persuaded the need of considering iVR interventions and other non-invasive medical devices with the same methodological design specificity we would treat comparing two different pharmacological agents, regarding the blinding process to reduce the risk of observer bias and distinguish between a placebo effect and the iVR effect [35].

Individuals suffering from bowel inflammatory disease can develop psychiatric symptoms such as anxiety or depression [36]. Among the three included studies, Çakır and Evirgen, 2021 avoided allocation bias by excluding patients diagnosed with psychiatric disorders (including anxiety disorders).

Regarding the role of the software content, we emphasise the single currently approved U.S. FDA cognitive-behavioural based software for chronic pain iVR, called EaseVRx versus a sham 2D nature video; both interventions were delivered through a Pico G2 4K resolution HMD. The study results show clear superiority in over-the-counter analgesics needed for pain control and sleep and physical function improvement in the EaseVRx group. However, patient opioid analgesics requirements were not significantly influenced in any of the two interventions [21]. The use of an HMD in both groups demonstrated the double-blind possibility of iVR. Regarding the intraprocedural colonoscopy studies presented, the lack of participant blinding can be counterweighted by implementing outcome assessor blinding.

### 4.1. iVR as an Alternative to Sedation during Colonoscopy

A 320 participant cross-sectional study using a survey assessed characteristics that influence the patient choice of iVR colonoscopy distraction instead of conventional sedated colonoscopy. Results revealed that anxiety was not related to willingness to accept VR colonoscopy (with a *p*-value of 0.686). One of the main reasons of the patients for not choosing VR colonoscopy as an alternative to conventional sedation was the expected procedure-induced pain (*p* = 0.025) [37].

Nature video content and lack of interaction through controllers may not be suitable for pain and anxiety reduction in colonoscopy interventions. It does not involve patients enough, limiting the sense of presence and being perceived more as an immersive television documentary than a true distractor. The principle of distracting patients with audio-visual stimuli during the colonoscopy was researched before iVR equipment, using DVD played auditory and video content during colonoscopy as a distraction. Still, although interventions lowered anxiety, their impact did not achieve statistical significance [38]. Historically, Lee et al., 2004, conducted a three-branch interventional study: the first group of enrolled participants were distracted only visually by watching a scenic-move home-made video through a Japanese-manufactured bidimensional (non-immersive) headset produced by Olympus, called EyeTrek; a second group received simultaneous visual and auditory stimulation while the control group received conventional sedation. Within the second audio-visually distracted group, a faster intervention completion time was achieved, along with greater patient satisfaction and willingness to undergo future procedures in the same manner and a lower dosage of analgesics needed to control sedation. Furthermore, the discussion section reveals that the investigators interpret their results by providing the most significant credit related to the distractibility potential to the auditory stimulation, not the add-on audio-visual cumulative effect [39]. The interesting finding was not according to reason, as following the add-on principle of immersivity, greater distractibility should be achieved through the synergic action of both stimuli. Still, 12 years later, the hypothesis of Lee et al., 2004 regarding auditory stimuli distractibility superiority was confirmed, when Silva et al., 2016, drew a similar conclusion after conducting a similar designed three-branch study, allowing participants to choose from a favourite song or movie; this time the individual distractibility potential of music vs. video exposure during colonoscopy was individually compared with a third conventional colonoscopy control group [20]. A possible logic of auditory stimuli superiority in distracting can be about the elective nature of the stimuli format, as the ability of humans to resonate with music exceeds the ease of emotional involvement compared with a video. The known medial pain pathway and amygdala associations coined a neuroanatomic premise of mediating pain perception through emotions. Still, it was only recently that Antioch et al., 2020 correlated exposure to favourite music with a rise of the pain threshold, presumably attributed to modulation of the anterior cingulate cortex [40]. Therefore, in correlation with preliminary research results and underlining the importance of auditory stimulation during colonoscopy [39,41]. Veldhuijzen et al., 2020 [30] RCT design might lower the level of immersion of participants by eliminating sound stimuli. Moreover, although satisfaction was high in the iVR group, the low-end iVR equipment reflected on the subjective sense of presence characteristics. The quality of the video immersion used created reasons for eight out of the ten participants in the VRG, four complaining about the lack of alternatives for the content, three about the quality of the movies and one was specific to complain was about the resolution. As all studies used low-end cardboard IVR powered by the Galaxy s7 smartphone, a shared sense of presence can be extrapolated to the other two studies. Thus, all the issues can influence the viewpoint of colonoscopy candidates. In other studies, low-end USD 15 HMDs such as Google Cardboard powered by smartphones was reported to lower pain perception [42], but the impact on anxiety was not among the outcomes.

Immersivity can be described as iVR HMD’s potential to offer a three-dimensional or stereoscopic perspective to the user, perceived as a “presence” in a computer-generated virtual environment. There is a direct proportionality between the level of sensory involvement and their quality, resulting in an illusion perceived as “presence” [43]. Presence within a virtual environment and higher levels of existent interaction feedback are the primary concepts that imbue the experience of the iVR user with affective, subjective meaning. Applying this concept lowers the perceived pain level through iVR and depends on the personal level of presence induced through immersivity, but also impacts individual psychological traits such as anxiety levels [44]. Distractibility occurs when the software-generated within the iVR HMD floods its user’s senses, offering a wide range of stimuli to process; if perceived vividly enough, an affective and motivational involvement will follow, known as presence, switching the prosexic function of his central nervous system away from reality [45]. Through distractibility, the user becomes less aware of the surrounding natural environment. It validates the fundamental aspect of VR’s applicability in surgical procedures by offering sanctuary to an anxious patient in a not-so-friendly hospital environment. Distractibility’s potential of lowering dimensions of perceived pain was observed in the case of severely burned patients exposed to a virtual environment during known painful procedures such as dressing change or physical therapy in both adult [46] and children populations [47], in some cases lowering patient anxiety and pain is realised to the point of unnecessary conventional analgesia [48].

Although how iVR exposure lowers the pain threshold has yet to be fully explained, one of the reasons may be attributed to the derealisation and depersonalisation potential of iVR exposure, a proven model of induced dissociation [49]. The hypothesis of dissociation as a post-traumatic survival psychological resort [50] can reflect in the patient experience in minimally invasive procedures, as iVR distractibility induced dissociation can lower the perception of pain from the primary focus of the user’s prosexic ability. The growing affordability of iVR equipment and consecutive ease of software development is associated with greater adoption of iVR within the medical world. Therefore, in theory, iVR could offer an interface for psychological interventions to optimise the cognitive and emotional status of patients involved in minimally invasive procedures such as colonoscopy-induced anxiety.

Using iVR HMD for sedation in colonoscopy is a financially more viable alternative. In France, a colonoscopy procedure is nearly three times more expensive when an anesthesiologist is involved [51]. Comparatively, although iVR HMD seems a one-time buy, dedicated software subscriptions are more and more affordable along with the widespread of the technology. A cost feasibility study analyses the savings for the hospitalised patient regarding pain treated by iVR versus conventional care was only USD 5.39 and rising to USD 98.49 savings per patient when the patient was both willing and eligible for iVR alternative treatment [52]. On the other hand, the main limitation of this study is that it is unicentric and studies the U.S. private health sector, one of the most costly health systems worldwide [53].

The most significant advantage of using iVR as an alternative to sedation is avoiding possible life-threatening adverse events associated with conventional sedation and analgesia, as seen in Table 4. On the other hand, the data extracted in Table 2 from the results section reveals that iVR during colonoscopy influenced anxiety and pain similar to conventional sedation and can be an alternative for patients unwilling to undergo sedation. None of the studies reported iVR intervention as the potential technical impediment regarding colonoscopy procedure, while patients in VRGs had an overall positive perception regarding the intervention. One of the main limitations of all studies is low-end iVR HMD. All three existing literature studies used more affordable HMD alternatives such as Google Cardboard, and two used Samsung Gear VR. In other studies, the use of low-end USD 15 HMDs such as Google Cardboard powered by smartphones was reported to lower pain perception [36], but the impact on anxiety was not one of the outcomes.

One of the most frequent side effects encountered after VR exposure is a form of motion sickness known as cybersickness. Cybersickness is influenced by predisposing traits such as gender but is also directly associated with certain hardware specifications such as interpupillary distance, the field of view, and image refresh rate [59]. Even new generation iVR HMD are prone to cybersickness weaknesses related to hardware specifications [60].

From a methodological point of view, among the three included studies, only Çakır and Evirgen, 2021, evaluated eligibility for exposure to a VR environment by excluding patients diagnosed with neurological disorders such as epilepsy or balance disorders [31]. The exclusion of cognitively impaired and dementia diagnosed patients was probably about to ensure conditions for valid measurement of pain through the VAS scale [61]. There is a strong need for a generalised ethical implementation within the current methodology of interventional iVR studies of preliminary patient evaluation through the Simulator Sickness Questionnaire (SSQ) [62].

Based on the reflected somatic impact of cybersickness, the documented resemblance to the somatic symptoms characterising acute anxiety events can be associated with higher perceived anxiety levels during the procedure. Individuals in the iVR group prone to simulator sickness can negatively perceive the immersive experience as physical symptoms such as dizziness, eye strain, and motion sickness over a stressful minimally invasive colonoscopy procedure. As only one study exposes the iVR group participant preliminary to procedure day, and that exposure did not assess cybersickness levels, nor was associated with procedural stress, a critical methodological issue might impair current studies designs. Currently, no existing literature directly correlates cybersickness similarities to a physical symptom of anxiety. However, many neuroscientist iVR interventional researchers stated that some SSQ items and anxiety traits are commonly encountered [63]. Anxiety can have a partial modulating effect on cybersickness within the immersive environment [64]. As all studies used low-end smartphone-powered iVR headsets, the chance of inducing cybersickness to predisposed individuals is further amplified, as the primary measurement of anxiety level is during a stressful, minimally invasive procedure such as colonoscopy. One of the aspects that can be influenced in controlling cybersickness is related to the type of iVR HMD specifications. Therefore, designing a well-calibrated interventional study could implement both pretesting newly emerged HMDs regarding their potential to induce cybersickness and also individual patient evaluation regarding SSQ scores before study inclusion is needed. Nonetheless, an attributed correction value before reporting results could be applied if participants are included without preliminary SSQ administration.

The evaluation of post-procedural anxiety does not consider that the person receiving the screening is most probably a colorectal cancer suspect or in a risk population such as inflammatory diseases. Perhaps the best moment for a proper evaluation of procedure-induced anxiety, diminishing the chance of measuring overlayed procedural pressure related to the result of the procedure, would be at a reasonable interval after receiving a precise diagnosis. Last but not least, among all minimally invasive medical or surgical procedures, as part of a screening program, colonoscopy candidates share vulnerable psychological traits.

### 4.2. iVR Education for Bowel Preparation in Reducing Anxiety before Colonoscopy

Regarding iVR patient education, a picture speaks more than 1000 words, but an iVR video might tell the whole story. Although a good bowel preparation was demonstrated to influence colonoscopy results by raising the chances of identifying a specific lesion and even spread intervals between re-examinations [65], more than a fifth of patients undergoing colonoscopy still lacks a proper preprocedural preparation [66], upcoming screening. Therefore, there is a growing need for offering better means of education to the patients before colonoscopy to influence the outcome measures related to the procedure. An iVR can raise patient motivation and provide a clear, less interpretable perspective regarding the procedural steps of colonoscopy. Explaining specific medical interventions is one of the fundamental ethical aspects of physicians worldwide, but sometimes it might be difficult as it implies rigid terminology or background knowledge. After years of practice, repetition of the exact instructions may be associated with a certain saturation level derived from routine; this may be reflected towards the patient as lack of interest, affecting non-verbal and paraverbal communication and leading to errors in transmitting specific vital instructions. The field of iVR given informed consent is both practical and resource-saving. The Surgical Planning and Informed Consent (SPLICE) results confirm the viability of iVR informed consent, although it did not lower STAI-measured anxiety significantly [67]. Perhaps the three-dimensional reconstructions of a standard virtual colonoscopy through software such as Surgical Theater™ can offer a truly immersive manner of informed consent to colonoscopy undergoing patients, acting as a psychological form of reassurance regarding the procedure. Interventions performed during colonoscopy (such as polypectomies or biopsies) can be described with optimal precision using immersive reconstruction techniques. This can represent an ethical milestone by facilitating the patient’s understanding of specific possible complications before consenting to colonoscopy. Also, it could offer the performing medical staff a possibility of pretraining according to the particular anatomical individuality of the patient within the iVR scenario before the procedure influencing performance anxiety towards improved outcomes.

Patients who found that the bowel preparation instructions were clear were much less anxious about the bowel preparation (15% with high anxiety versus 36% among those who thought the instructions were confusing) [12].

Liu Z. et al., 2017, offered a panoramic view over the worldwide encountered forms of education in colonoscopy bowel preparation, underlining the disadvantages of printed materials that do not apply in patient education or the time consuming and variability of the information offered through counselling and training sessions. Audio-visual, smartphone, and social media-based education are restricted to developed areas [68].

Recommendations for patient education regarding bowel preparation would be represented by portable autonomous iVR HMDs. Such as Oculus Go or Oculus Quest with pre-programmed training videos that may include knowledge testing. A viable alternative for patient bowel preparation is represented by pre-programmed autonomous standalone iVR HMD devices (such as Oculus Go, Oculus Quest 1 and 2) backed by dedicated educational software.

The current advancement of iVR in education relies on being perceived as a tool for adapting the social distancing during the SARS-CoV-2 pandemic; this exponential growth trend may continue beyond the end of the restrictions. An iVR education for bowel preparation can raise patient motivation and satisfaction and offer a more precise, less interpretable perspective regarding the procedural steps of colonoscopy. Improving satisfaction and self-reported anxiety levels can improve better screening cooperation.

### 4.3. How Colonoscopy Can Progress through the Implementation of iVR

Colonoscopy is one of the most performed minimally invasive procedures, part of national screening programs for colon cancer (the United Kingdom’s Colorectal Cancer Screening Programme or the Colorectal Cancer Control Program in the United States of America). Over 14.2 million colonoscopies and 2.8 million flexible sigmoidoscopies were performed in the U.S. during 2002 [69], with a similar number of colonoscopies but more than double sigmoidoscopies performed in 2013 [70].

The pandemic aftermath weighs heavily on any health system, extending the gap between the healthcare professionals and patients with a risk for colon cancer redistributing resources away from screening programs [71].

The anxiety related to the possibility of contracting SARS-CoV-2 has a cumulative effect reflected in a lower screening of the risk population [72]. Colonoscopy anxiety influences the results of the procedure and the chance of failure to attend to scheduled interventions. The outcome measuring the magnitude of anxiety in patients regarding colonoscopy was systematically reviewed by Yang et al., 2018, and according to the 14 included studies that used the Spielberger STAI-T psychometric scale (scored from 10 to 80) to measure anxiety levels on the day of the procedure, reported results of mean anxiety scores ranged from 35.5 to 46.9. Regarding a comparison between anxiety a day before the procedure and during procedure day, only two of the studies included in the review shared this specific outcome, documenting a statistically significant increase in the latter, from a median of 29.1 to 32.5 STAI-S (*p* = 0.05) range a day before to a median anxiety score ranging from 36.2 to 44.8 (*p* = 0.001) on colonoscopy procedure day anxiety [13].

Complementary fields of minimally invasive medical procedures and modalities such as burnt patient debridement have more than a decade since proven to benefit from experimenting with computer-simulated VR exposure [73]. Colonoscopy iVR implementation regarding this approach is emerging but seems not fully committed to state-of-the-art interventional equipment. Immersion quality induced by HMD was up to 34% in more experienced high-end equipment. Another important aspect is that the percentage was reported regarding the technological level development of 2006. The study also reports further lowering of psychological elements of mentally focusing on pain by 29%, and an add-on 46% reduction in pain unpleasantness [74], long before the milestones associated with iVR’s “breaking through the wall” of a seven-decade technological marathon, to finally becoming qualitative enough to trick our central nervous system and even compete with reality. Muñoz-Saavedra et al., 2020, reported an increasing publication trend regarding iVR-facilitated laparoscopic minimally invasive procedures in Europe [75], so although progress towards colonoscopy use with high-end iVR is slow, it is proceeding

The level of immersivity is directly correlated with reducing pain. Still, technological development speed outruns our ability to discern state-of-the-art hardware worthy of medical interventions from outdated iVR devices. Perhaps the rate of progress is more than we can process considering the preparations regarding a clinical trial; there is a chance that the equipment used will outdate before starting the study. Therefore, outdated technology is far more dangerous, reflected in the perpetual lack of correctly differentiating computer-simulated VR environments and true state-of-the-art iVR. This could directly impact progress towards implementing an already insufficiently acknowledged disruptive technology such as iVR. The results may trigger lesser enthusiasm in researchers in replicating interventional with targeted outcomes that do not obey the immovable weight of the statistically insignificant “*p*”. Similar methodological flawed designs and results were reported in other cancer screening minimally invasive procedures such as iVR cystoscopy distraction for 23 CG vs. 22 iVR group [76]. According to Ronald Fisher, “A scientific fact should be regarded as experimentally established only if a properly designed experiment rarely fails to provide this level of significance.” [77].

Jae-Hyuk Yang, 2021, studied the impact of a high-end HTC Vive headset and content specific to the following arthroscopic reconstruction procedural success instead of random natural environments lowered anxiety scores by possibly targeting a psychological reassurance mechanism. This aspect can be explained by acknowledging the distractibility potential of iVR on nociceptive stimuli in both immersive and non-immersive exposure; therefore, implying that quality immersion manages to reach a level of distractibility that impairs internal cognitive processes involved in anxiety mechanisms [78].

Finally, physicians performing invasive or minimally invasive procedures such as colonoscopy training may benefit from iVR exposure training by lowering performance anxiety. The improvement of hand coordination [79], greater procedural confidence [80], facilitation of the acquisition of complex skills and also translating learned aspects into practice was reported to be even five times as efficient compared with conventional training methods [81]. To date, no colonoscopy simulators offer iVR compatible interfaces.

According to the definition of iVR by Grigore Burdea, it must resemble “a high-end user-computer interface that involves real-time simulation and interactions through multiple sensorial channels” [82]. Considering the tremendous technological disruptions regarding this domain, it is essential to ensure the use of state-of-the-art HMD equipment and software in the research process. Most VR interventional studies do not differentiate immersive virtual reality (HMD) from the bi-dimensional display that most medical simulators still encounter. However, there is a clear gap in the level of immersivity and distractibility induced. Moreover, in recent studies, immersivity of virtual reality is based on outdated hardware equipment consisting of cardboard headsets associated with smartphones, having clear limitations compared with dedicated state-of-the-art headsets regarding the quality of immersivity of visual stimuli (pixel density), refresh rate (frames per second), and the field of view (in horizontal and vertical degrees). A higher level of anxiety is correlated with a more robust perception of pain for patients and an impaired procedural efficiency of the performing medical staff.

The unique qualitative benefits of iVR implementation in colonoscopy as a sedation alternative, interface for offering patients bowel preparation education, and perhaps facilitating translation of physician simulator training to clinical practice can be cumulative if simultaneously applied in all three aspects. Therefore, raising the bar for full integration of high-end iVR in colonoscopy can progressively benefit the impact of individual interventions by accommodating both medical staff and patients to use and provide feedback regarding the promotion of this disruptive technology towards its potential.

Limitations: As an emerging medical technology, there are few published iVR interventional studies in colonoscopy that significantly differ methodologically even when targeting the same populations, interventions, outcomes and using similar hardware tools. The current small sample sizes emphasise the need for more research to ensure the validity of the iVR approach targeting colonoscopy-induced anxiety and pain. In the absence of a commonly approved framework, the pursuit of outcomes is based on what directions the investigators consider worthy. This aspect leads to heterogenous gathered results, less poolable for relevant statistical analysis. The iVR techniques’ potential has been recently evaluated for colonoscopy anxiety; the novelty of the research topic is associated with only a handful of currently published papers.

## 5. Conclusions

Current clinical trials regarding intraprocedural use of iVR for colonoscopy-induced anxiety and pain share the common methodological flaw of using low-end iVR and non-interactive software. Intraprocedural iVR interventions for colonoscopy-induced anxiety and pain reveal superiority to non-sedated colonoscopy and share similar effects compared to sedated colonoscopy. Future studies should have common frameworks to gather homogenous data for statistical analysis. Directions of study designs regarding the impact of iVR on anxiety should optimise the eligibility of participants, double-blinding process, randomization and SSQ implementation.

An iVR patient education for bowel preparation represents a promising educational tool that lowers the preprocedural anxiety of the patient.

iVR HMD interventions have the potential to become an alternative for anxiety and pain management in colonoscopy, especially when sedation is undesired or specifically contraindicated. However, more research is needed regarding this field.

## Figures and Tables

**Table 1:** Search strategy.

Theme	MeSH Descriptor and Boolean Descriptors	Keywords and Boolean Descriptors
Virtual reality	[Virtual Reality Exposure Therapy] OR [Smart Glasses] OR [Virtual Reality]	VR OR “virtual therapy” OR “virtual environment” OR “virtual treatment” OR “immersive” OR “non-immersive” OR hmd OR “head-mounted display” OR HTC OR oculus
Colonoscopy	[Colonoscopy] OR [Sigmoidoscopy] OR [Endoscopy]	colonoscopy OR sigmoidoscopy OR rectoscope OR endoscopy OR “gastrointestinal endoscopy” OR “secondary prevention” OR prevention
Anxiety/pain/sedative/anxiolytic therapy	[Pain] OR [Fear] OR [Anxiety] OR [Heart Rate] OR [Blood Pressure] OR [Anti-Anxiety Agents] OR [Antidepressive Agents] OR [Barbiturates] OR [Benzodiazepines] OR [Hypnotics and Sedatives]	“pain score” OR “pain level” OR fears OR “fear score” OR “fear level” OR “anxiety level” OR “anxiety score” OR pulse OR “pulse rate”

**Table 2:** Current interventional studies that evaluate iVR effect on colonoscopy intraprocedural induced anxiety and pain.

Authors	RCT Design; Participants (n)	iVR Intervention: Hardware, Software, Duration	Psychometric Evaluation of Anxiety				Pain Evaluation			
Veldhuijzen et al., 2020 [30]	RCT VRG: (10), CG: (9) Sedated	Samsung Gear VR (Consumer Edition–SM-R322), powered by a Samsung Galaxy S7 smartphone running: A mute 19 min and 59 s long 360° video of tropical islands and forests in the Caribbean.	STAI-S and STAI-T				NRS			
				VRG	CG	*p*		VRG	CG	*p*
			STAI-S Pre:	48.5	49	0.497	Pre:	0 (0, 3)	0 (0, 1.75)	0.968
			STAI-S Post:	50	50	0.156	During:	3 (1.5, 5.5)	3 (1, 4)	0.661
			STAI-T:	29	35	0.549				
Çakır and Evirgen, 2021 [31]	RCT VRG: (30), CG: (30) Non-sedated	VR Cardboard Super Flex Googles Android smartphone-powered headset running: “A walk on the beach” software (2016) for the entire procedure duration.	STAI-T and STAI-S				VAS (1–10)			
				VRG	CG	*p*		VRG	CG	*p*
			STAI-T:	39.73 ± 3.14	46.70 ± 5.97	0.000	During:	2.76 ± 1.25	3.76 ± 2.11	0.03
			STAI-S Pre:	47.70 ± 3.55	48.28 ± 5.26	0.647	Post:	0.83 ± 1.44	1.36 ± 1.51	0.168
			STAI-S Post:	46.83 ± 10.94	49.66 ± 2.83	0.175				
Friedman et al., 2021 [32]	Pilot study Comparing iVR experience of previously consciously sedated patients VRG (27)	Samsung Gear VR Headset powered by Samsung Galaxy S7 smartphone running: 1 to 7-min video of nature and animal content, starting synchronously with colonoscopy procedure.	1–10 patient-reported questionnaires				VAS (1–10)			
			Pre-procedure anxiety mean ± SD: 3.8 ± 2.2				Procedural pain mean: 3.6 ± 1.6			
			Anxiety vs. past sedation colonoscopy				Pain vs. past colonoscopy			
			Less: 11 (42.3%)				Less: 1 (3.8-requested sedation),			
			Similar: 8 (30.8%)				Similar: 11 (42.3)			
			More: 3 (11.5%)				More:11 (42.3)			
			Not remembering: 1 (3.8%)				No response: 3 (11.5)			

Data are reported as mean ± SD. Randomized control trial (RCT), iVR group (VRG), control group (CG); Spielberger State-Trait Anxiety Inventory (20–80): STAI-S and STAI-T; the Visual Analog Scale (VAS) ranging from 1 cm to 10 cm (1–10), Numeric Pain Rating Scale (NRS), standard deviation (SD) Student’s *t*-test (*p*).

**Table 3:** iVR RCT education for bowel preparation impacts patient anxiety levels and preparation efficacy.

Author	Study Design Enrolled Participants (*n*)	Outcomes	Anxiety Levels Self-Rated Night before Colonoscopy Sleep Quality	The Boston Bowel Preparation Scale (0–9)
Chen et al., 2021 [33]	Single-centre prospective, single-blinded, RCT (346) undergoing the first colonoscopy 173 VRG 173 CG	Primary: evaluate the feasibility of iVR HMD videos for bowel preparation patient education before colonoscopy by comparing them to conventional schooling. Secondary: preprocedural anxiety, adenoma, and polyp detection rates, compliance to bowel cleansing, satisfaction, and willingness to undergo future recommended colonoscopies.	Anxiety score: VRG: 7.60 (2.20) CG: 7.08 (2.64) *p*: 0.04	Mean score of ascending, transverse, and descending colon: VRG: 7.61 (1.65) CG: 7.04 (1.70) *p*: 0.002
				Adequate bowel preparation: VRG: 139 (80.3) CG: 125 (72.3) *p*: 0.08

Data are presented as mean (SD). Randomized control trial (RCT), iVR group (VRG), control group (CG).

**Table 4:** Comparison of side effects of sedation colonoscopy vs. iVR exposure complications.

	Sedation and Analgesia Colonoscopy Complications [54]	Immersive Virtual Reality Side Effects [55,56,57,58]
Cardiovascular:	Hypotension, hypertension, arrhythmias, myocardial ischemia/infarction	-
Respiratory:	Decreased breathing rate, respiratory depression, airway obstruction, hypoxia, pulmonary aspiration	-
Allergic reactions:	Minor local response to anaphylactic reactions	-
Central nervous system:	Paradoxical reactions (benzodiazepines)	Theoretical epileptic seizures related to intermittent light stimuli in children [55] static imbalance: dizziness, eye strain [56] dissociative symptoms: depersonalization/derealization disorder [57]
Other:	Analgesic induced nausea, vomiting	Cybersickness [58]: 3rd cranial nerve (headache, eyestrain, blurred vision) disorientation (imbalance, vertigo) nausea (vomiting, dizziness).

## References

[1] Bhise V., Modi V., Kalavar A., Espadas D., Hanser L., Gould M., El-Serag H.B., Singh H. (2016). Patient-Reported Attributions for Missed Colonoscopy Appointments in Two Large Healthcare Systems. Dig. Dis. Sci..

[2] Braithwaite E., Carbonell J., Kane J.S., Gracie D., Selinger C.P. (2021). Patients’ Perception of Colonoscopy and Acceptance of Colonoscopy Based IBD Related Colorectal Cancer Surveillance. Expert Rev. Gastroenterol. Hepatol..

[3] Shafer L.A., Walker J.R., Waldman C., Yang C., Michaud V., Bernstein C.N., Hathout L., Park J., Sisler J., Restall G. (2018). Factors Associated with Anxiety About Colonoscopy: The Preparation, the Procedure, and the Anticipated Findings. Dig. Dis. Sci..

[4] Aljebreen A.M., Almadi M.A., Leung F.W. (2014). Sedated vs Unsedated Colonoscopy: A Prospective Study. World J. Gastroenterol. WJG.

[5] Tran T.N., Ferrari A., Hoeck S., Peeters M., Van Hal G. (2021). Colorectal Cancer Screening: Have We Addressed Concerns and Needs of the Target Population?. Gastrointest. Disord..

[6] Levin T.R. (2017). Beyond Colonoscopy: The Role of Alternative Screening Tests for Colorectal Cancer in Your Practice. Am. J. Gastroenterol..

[7] Sung H., Ferlay J., Siegel R.L., Laversanne M., Soerjomataram I., Jemal A., Bray F. (2021). Global Cancer Statistics 2020: GLOBOCAN Estimates of Incidence and Mortality Worldwide for 36 Cancers in 185 Countries. CA Cancer J. Clin..

[8] Ferlizza E., Solmi R., Sgarzi M., Ricciardiello L., Lauriola M. (2021). The Roadmap of Colorectal Cancer Screening. Cancers.

[9] Anghel S.A., Ioniță-Mîndrican C.-B., Luca I., Pop A.L. (2021). Promising Epigenetic Biomarkers for the Early Detection of Colorectal Cancer: A Systematic Review. Cancers.

[10] Ferrari A., Neefs I., Hoeck S., Peeters M., Van Hal G. (2021). Towards Novel Non-Invasive Colorectal Cancer Screening Methods: A Comprehensive Review. Cancers.

[11] Spada C., Hassan C., Bellini D., Burling D., Cappello G., Carretero C., Dekker E., Eliakim R., de Haan M., Kaminski M.F. (2020). Imaging Alternatives to Colonoscopy: CT Colonography and Colon Capsule. European Society of Gastrointestinal Endoscopy (ESGE) and European Society of Gastrointestinal and Abdominal Radiology (ESGAR) Guideline—Update 2020. Endoscopy.

[12] Suffeda A., Meissner W., Rosendahl J., Guntinas-Lichius O. (2016). Influence of Depression, Catastrophizing, Anxiety, and Resilience on Postoperative Pain at the First Day after Otolaryngological Surgery. Medicine.

[13] Yang C., Sriranjan V., Abou-Setta A.M., Poluha W., Walker J.R., Singh H. (2018). Anxiety Associated with Colonoscopy and Flexible Sigmoidoscopy: A Systematic Review. Am. J. Gastroenterol..

[14] Hassan C., Antonelli G. (2020). I Want to Have Virtual Reality Distraction during My Colonoscopy!. Endosc. Int. Open.

[15] Gams M., Kolenik T. (2021). Relations between Electronics, Artificial Intelligence and Information Society through Information Society Rules. Electronics.

[16] Xiong J., Hsiang E.-L., He Z., Zhan T., Wu S.-T. (2021). Augmented Reality and Virtual Reality Displays: Emerging Technologies and Future Perspectives. Light Sci. Appl..

[17] Sutherland J., Belec J., Sheikh A., Chepelev L., Althobaity W., Chow B.J.W., Mitsouras D., Christensen A., Rybicki F.J., La Russa D.J. (2019). Applying Modern Virtual and Augmented Reality Technologies to Medical Images and Models. J. Digit. Imaging.

[18] Botella C., Baños R.M., García-Palacios A., Quero S. (2017). Virtual Reality and Other Realities. The Science of Cognitive Behavioral Therapy.

[19] Hersh A., Mahapatra S., Weber-Levine C., Awosika T., Theodore J.N., Zakaria H.M., Liu A., Witham T.F., Theodore N. (2021). Augmented Reality in Spine Surgery: A Narrative Review. HSS J. Musculoskelet. J. Hosp. Spec. Surg..

[20] Basso A. (2017). Advantages, Critics and Paradoxes of Virtual Reality Applied to Digital Systems of Architectural Prefiguration, the Phenomenon of Virtual Migration. Proceedings.

[21] Garcia L.M., Birckhead B.J., Krishnamurthy P., Sackman J., Mackey I.G., Louis R.G., Salmasi V., Maddox T., Darnall B.D. (2021). An 8-Week Self-Administered At-Home Behavioral Skills-Based Virtual Reality Program for Chronic Low Back Pain: Double-Blind, Randomized, Placebo-Controlled Trial Conducted During COVID-19. J. Med. Internet Res..

[22] Tamang L.D., Kim B.W. (2021). Deep Learning Approaches to Colorectal Cancer Diagnosis: A Review. Appl. Sci..

[23] Rauschnabel P.A. (2021). Augmented Reality Is Eating the Real-World! The Substitution of Physical Products by Holograms. Int. J. Inf. Manag..

[24] Kılıç A., Brown A., Aras I., Hui R., Hare J., Hughes L.D., McCracken L.M. (2021). Using Virtual Technology for Fear of Medical Procedures: A Systematic Review of the Effectiveness of Virtual Reality-Based Interventions. Ann. Behav. Med. Publ. Soc. Behav. Med..

[25] Georgescu R., Fodor L.A., Dobrean A., Cristea I.A. (2020). Psychological Interventions Using Virtual Reality for Pain Associated with Medical Procedures: A Systematic Review and Meta-Analysis. Psychol. Med..

[26] Eijlers R., Dierckx B., Staals L.M., Berghmans J.M., van der Schroeff M.P., Strabbing E.M., Wijnen R.M.H., Hillegers M.H.J., Legerstee J.S., Utens E.M.W.J. (2019). Virtual Reality Exposure before Elective Day Care Surgery to Reduce Anxiety and Pain in Children: A Randomized Controlled Trial. Eur. J. Anaesthesiol..

[27] Nordgård R., Låg T. (2021). The Effects of Virtual Reality on Procedural Pain and Anxiety in Pediatrics: A Systematic Review and Meta-Analysis. Front. Virtual Real..

[28] Koo C.-H., Park J.-W., Ryu J.-H., Han S.-H. (2020). The Effect of Virtual Reality on Preoperative Anxiety: A Meta-Analysis of Randomized Controlled Trials. J. Clin. Med..

[29] Moher D., Liberati A., Tetzlaff J., Altman D.G. (2009). Preferred Reporting Items for Systematic Reviews and Meta-Analyses: The PRISMA Statement. BMJ.

[30] Veldhuijzen G., Klaassen N.J.M., Van Wezel R.J.A., Drenth J.P.H., Van Esch A.A. (2020). Virtual Reality Distraction for Patients to Relieve Pain and Discomfort during Colonoscopy. Endosc. Int. Open.

[31] Cakir S.K., Evirgen S. (2021). The Effect of Virtual Reality on Pain and Anxiety During Colonoscopy: A Randomized Controlled Trial. Turk. J. Gastroenterol..

[32] Friedman M., Rand K., Patel T., Colizzo F., Carolan P., Kelsey P., Chung D.C. (2021). A Pilot Study of Virtual Reality as an Alternative to Pharmacological Sedation during Colonoscopy. Endosc. Int. Open.

[33] Chen G., Zhao Y., Xie F., Shi W., Yang Y., Yang A., Wu D. (2021). Educating Outpatients for Bowel Preparation Before Colonoscopy Using Conventional Methods vs Virtual Reality Videos Plus Conventional Methods: A Randomized Clinical Trial. JAMA Netw. Open.

[34] Le May S., Tsimicalis A., Noel M., Rainville P., Khadra C., Ballard A., Guingo E., Cotes-Turpin C., Addab S., Chougui K. (2021). Immersive Virtual Reality vs. Non-immersive Distraction for Pain Management of Children during Bone Pins and Sutures Removal: A Randomized Clinical Trial Protocol. J. Adv. Nurs..

[35] Dualé C., Mourgues C. (2022). The Price of Pain Relief, or Should Non-Invasive Medical Devices Be Treated Differently in Analgesic Clinical Trials?. Eur. J. Pain.

[36] Choi K., Chun J., Han K., Park S., Soh H., Kim J., Lee J., Lee H.J., Im J.P., Kim J.S. (2019). Risk of Anxiety and Depression in Patients with Inflammatory Bowel Disease: A Nationwide, Population-Based Study. J. Clin. Med..

[37] Blokzijl S.J., Lamberts K.F., van der Waaij L.A., Spikman J.M. (2019). Willingness to Undergo Colonoscopy with Virtual Reality Instead of Procedural Sedation and Analgesia. Eur. J. Gastroenterol. Hepatol..

[38] Xiaolian J., Xiaolin L., Lan Z.H. (2015). Effects of Visual and Audiovisual Distraction on Pain and Anxiety Among Patients Undergoing Colonoscopy. Gastroenterol. Nurs..

[39] Lee D.W.H., Chan A.C.W., Wong S.K.H., Fung T.M.K., Li A.C.N., Chan S.K.C., Mui L.M., Ng E.K.W., Chung S.C.S. (2004). Can Visual Distraction Decrease the Dose of Patient-Controlled Sedation Required During Colonoscopy? A Prospective Randomized Controlled Trial. Endoscopy.

[40] Antioch I., Furuta T., Uchikawa R., Okumura M., Otogoto J., Kondo E., Sogawa N., Ciobica A., Tomida M. (2020). Favorite Music Mediates Pain-Related Responses in the Anterior Cingulate Cortex and Skin Pain Thresholds. J. Pain Res..

[41] Silva A.P.D., Niriella M.A., Nandamuni Y., Nanayakkara S.D., Perera K.R.P., Kodisinghe S.K., Subasinghe K.C.E., Pathmeswaran A., Silva H.J. (2016). de Effect of Audio and Visual Distraction on Patients Undergoing Colonoscopy: A Randomized Controlled Study. Endosc. Int. Open.

[42] Patel P., Ivanov D., Bhatt S., Mastorakos G., Birckhead B., Khera N., Vittone J. (2020). Low-Cost Virtual Reality Headsets Reduce Perceived Pain in Healthy Adults: A Multicenter Randomized Crossover Trial. Games Health J..

[43] Slater M. (2018). Immersion and the Illusion of Presence in Virtual Reality. Br. J. Psychol..

[44] Triberti S., Repetto C., Riva G. (2014). Psychological Factors Influencing the Effectiveness of Virtual Reality-Based Analgesia: A Systematic Review. Cyberpsychol. Behav. Soc. Netw..

[45] Găină M.-A., Boloș A., Alexinschi O., Cristofor A.-C., Găină A.-M., Chiriță R., Ștefănescu C. (2021). Perspective on the Double Edges of Virtual Reality in Medicine—Both Addiction & Treatment. BRAIN Broad Res. Artif. Intell. Neurosci..

[46] Luo H., Cao C., Zhong J., Chen J., Cen Y. (2019). Adjunctive Virtual Reality for Procedural Pain Management of Burn Patients during Dressing Change or Physical Therapy: A Systematic Review and Meta-Analysis of Randomized Controlled Trials: Virtual Reality for Burn Patients: A Systematic Review and Meta-Analysis of Randomized Controlled Trials. Wound Repair Regen..

[47] Lauwens Y., Rafaatpoor F., Corbeel K., Broekmans S., Toelen J., Allegaert K. (2020). Immersive Virtual Reality as Analgesia during Dressing Changes of Hospitalized Children and Adolescents with Burns: A Systematic Review with Meta-Analysis. Children.

[48] Mallari B., Spaeth E.K., Goh H., Boyd B.S. (2019). Virtual Reality as an Analgesic for Acute and Chronic Pain in Adults: A Systematic Review and Meta-Analysis. J. Pain Res..

[49] Aardema F., O’Connor K., Côté S., Taillon A. (2010). Virtual Reality Induces Dissociation and Lowers Sense of Presence in Objective Reality. Cyberpsychol. Behav. Soc. Netw..

[50] Schauer M., Elbert T. (2010). Dissociation Following Traumatic Stress: Etiology and Treatment. Z. Psychol. Psychol..

[51] Hassan C., Benamouzig R., Spada C., Ponchon T., Zullo A., Saurin J.C., Costamagna G. (2011). Cost Effectiveness and Projected National Impact of Colorectal Cancer Screening in France. Endoscopy.

[52] Delshad S.D., Almario C.V., Fuller G., Luong D., Spiegel B.M.R. (2018). Economic Analysis of Implementing Virtual Reality Therapy for Pain among Hospitalized Patients. npj Digit. Med..

[53] Papanicolas I., Woskie L.R., Jha A.K. (2018). Health Care Spending in the United States and Other High-Income Countries. JAMA.

[54] Amornyotin S. (2013). Sedation-Related Complications in Gastrointestinal Endoscopy. World J. Gastrointest. Endosc..

[55] Tychsen L., Thio L.L. (2020). Concern of Photosensitive Seizures Evoked by 3D Video Displays or Virtual Reality Headsets in Children: Current Perspective. Eye Brain.

[56] Park S., Lee G. (2020). Full-Immersion Virtual Reality: Adverse Effects Related to Static Balance. Neurosci. Lett..

[57] Spiegel J.S. (2018). The Ethics of Virtual Reality Technology: Social Hazards and Public Policy Recommendations. Sci. Eng. Ethics.

[58] Weech S., Kenny S., Barnett-Cowan M. (2019). Presence and Cybersickness in Virtual Reality Are Negatively Related: A Review. Front. Psychol..

[59] Stanney K., Fidopiastis C., Foster L. (2020). Virtual Reality Is Sexist: But It Does Not Have to Be. Front. Robot. AI.

[60] Caserman P., Garcia-Agundez A., Gámez Zerban A., Göbel S. (2021). Cybersickness in Current-Generation Virtual Reality Head-Mounted Displays: Systematic Review and Outlook. Virtual Real..

[61] Breivik H., Borchgrevink P.C., Allen S.M., Rosseland L.A., Romundstad L., Hals E.K.B., Kvarstein G., Stubhaug A. (2008). Assessment of Pain. Br. J. Anaesth..

[62] Găină M.-A., Ștefănescu C. The Urge for an Ethical Framework Regarding Virtual Reality Interventional Studies in Psychiatry. Proceedings of the Book International Congress of Bioethics.

[63] Bouchard S., Berthiaume M., Robillard G., Forget H., Daudelin-Peltier C., Renaud P., Blais C., Fiset D. (2021). Arguing in Favor of Revising the Simulator Sickness Questionnaire Factor Structure When Assessing Side Effects Induced by Immersions in Virtual Reality. Front. Psychiatry.

[64] Pot-Kolder R., Veling W., Counotte J., van der Gaag M. (2018). Anxiety Partially Mediates Cybersickness Symptoms in Immersive Virtual Reality Environments. Cyberpsychol. Behav. Soc. Netw..

[65] Lebwohl B., Kastrinos F., Glick M., Rosenbaum A.J., Wang T., Neugut A.I. (2011). The Impact of Suboptimal Bowel Preparation on Adenoma Miss Rates and the Factors Associated with Early Repeat Colonoscopy. Gastrointest. Endosc..

[66] Liu X., Luo H., Zhang L., Leung F.W., Liu Z., Wang X., Huang R., Hui N., Wu K., Fan D. (2014). Telephone-Based Re-Education on the Day before Colonoscopy Improves the Quality of Bowel Preparation and the Polyp Detection Rate: A Prospective, Colonoscopist-Blinded, Randomized, Controlled Study. Gut.

[67] Perin A., Galbiati T.F., Ayadi R., Gambatesa E., Orena E.F., Riker N.I., Silberberg H., Sgubin D., Meling T.R., DiMeco F. (2021). Informed Consent through 3D Virtual Reality: A Randomized Clinical Trial. Acta Neurochir..

[68] Liu Z., Zhang M.M., Li Y.Y., Li L.X., Li Y.Q. (2017). Enhanced Education for Bowel Preparation before Colonoscopy: A State-of-the-Art Review. J. Dig. Dis..

[69] Vijan S., Inadomi J., Hayward R.A., Hofer T.P., Fendrick A.M. (2004). Projections of Demand and Capacity for Colonoscopy Related to Increasing Rates of Colorectal Cancer Screening in the United States. Aliment. Pharmacol. Ther..

[70] Joseph D.A., Meester R.G.S., Zauber A.G., Manninen D.L., Winges L., Dong F.B., Peaker B., van Ballegooijen M. (2016). Colorectal Cancer Screening: Estimated Future Colonoscopy Need and Current Volume and Capacity. Cancer.

[71] Harber I., Zeidan D., Aslam M.N. (2021). Colorectal Cancer Screening: Impact of COVID-19 Pandemic and Possible Consequences. Life.

[72] Mazidimoradi A., Tiznobaik A., Salehiniya H. (2021). Impact of the COVID-19 Pandemic on Colorectal Cancer Screening: A Systematic Review. J. Gastrointest. Cancer.

[73] Hoffman H.G., Chambers G.T., Meyer W.J., Arceneaux L.L., Russell W.J., Seibel E.J., Richards T.L., Sharar S.R., Patterson D.R. (2011). Virtual Reality as an Adjunctive Non-Pharmacologic Analgesic for Acute Burn Pain during Medical Procedures. Ann. Behav. Med. Publ. Soc. Behav. Med..

[74] Hoffman H.G., Seibel E.J., Richards T.L., Furness T.A., Patterson D.R., Sharar S.R. (2006). Virtual Reality Helmet Display Quality Influences the Magnitude of Virtual Reality Analgesia. J. Pain.

[75] Muñoz-Saavedra L., Miró-Amarante L., Domínguez-Morales M. (2020). Augmented and Virtual Reality Evolution and Future Tendency. Appl. Sci..

[76] Walker M.R., Kallingal G.J.S., Musser J.E., Folen R., Stetz M.C., Clark J.Y. (2014). Treatment Efficacy of Virtual Reality Distraction in the Reduction of Pain and Anxiety during Cystoscopy. Mil. Med..

[77] Curran-Everett D. (2020). Evolution in Statistics: P Values, Statistical Significance, Kayaks, and Walking Trees. Adv. Physiol. Educ..

[78] Yang J.-H., Ryu J.J., Nam E., Lee H.-S., Lee J.K. (2019). Effects of Preoperative Virtual Reality Magnetic Resonance Imaging on Preoperative Anxiety in Patients Undergoing Arthroscopic Knee Surgery: A Randomized Controlled Study. Arthrosc. J. Arthrosc. Relat. Surg..

[79] Martirosov S., Hořejší P., Kopeček P., Bureš M., Šimon M. (2021). The Effect of Training in Virtual Reality on the Precision of Hand Movements. Appl. Sci..

[80] Pulijala Y., Ma M., Pears M., Peebles D., Ayoub A. (2018). Effectiveness of Immersive Virtual Reality in Surgical Training—A Randomized Control Trial. J. Oral Maxillofac. Surg..

[81] Lohre R., Bois A.J., Athwal G.S., Goel D.P., Canadian Shoulder and Elbow Society (CSES) (2020). Improved Complex Skill Acquisition by Immersive Virtual Reality Training: A Randomized Controlled Trial. J. Bone Jt. Surg. Am..

[82] Coiffet P., Burdea G.C. (2017). Virtual Reality Technology.

